# Supplementary material for: Gastric Microbiota in Helicobacter pylori-Negative and -Positive Gastritis Among High Incidence of Gastric Cancer Area
Source: Cancers (Basel). 2019 Apr 10;11(4):504. doi: 10.3390/cancers11040504 (PMC6520852; doi:10.3390/cancers11040504)
Supplement: Supplementary file 1 [file cancers-11-00504-s001.pdf]

Supplementary Material:

Gastric Microbiota in *Helicobacter pylori*-Negative and -Positive Gastritis Among High Incidence of Gastric Cancer Area

Boldbaatar Gantuya, Hashem B. El-Serag, Takashi Matsumoto, Nadim J. Ajami, Khasag Oyuntsetseg, Dashdorj Azzaya, Tomohisa Uchida and Yoshio Yamaoka

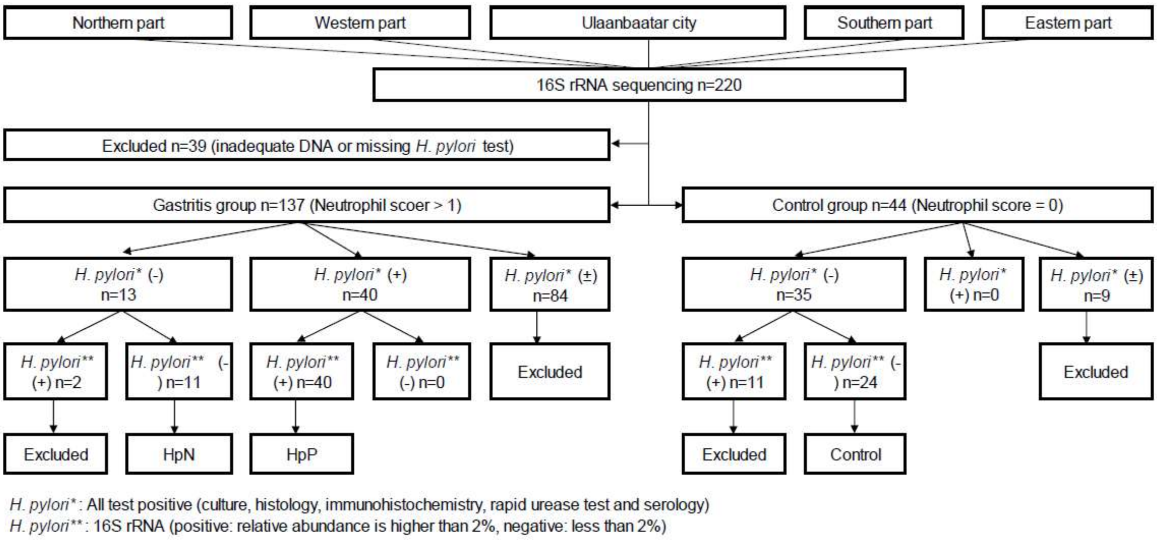

Figure S1. Flow chart for case selection in the three study groups. The case selection is based on neutrophil infiltration and *H. pylori* infection status. For gastritis diagnosis, any gastric site (antrum, corpus and incisura angularis) with neutrophil infiltration score ≥1 is considered gastritis. For *H. pylori* diagnosis, *H. pylori**: gold standard method based on culture, histology, immunohistochemistry and serological method; *H. pylori***: 16S rRNA approach based on relative abundance (RA). RA ≥ 2% of *H. pylori* considered positive and <2% as negative. From overall cases 39 were excluded because of suboptimal DNA quantity. Of the remaining 181 cases, 137 had gastritis based on PMN score ≥1 presence in any gastric biopsy site, and the other 44 had no gastritis with PMN score < 1. Of the 181 cases, 133 had positive testing for *H. pylori* on at least one of the five conventional tests, and 48 were negative for all five tests. Of those, we selected only cases with positive results for *all* five conventional tests (*n* = 40) for further analysis as *H. pylori*-positive cases and excluded the rest (*n* = 93). All groups were further evaluated for *H. pylori* status by 16S rRNA gene sequencing using a relative abundance cut off value of 2% for positive results based on previous study. All 40 *H. pylori*-positive gastritis (HpP) cases were confirmed positive by 16S rRNA gene sequencing. Of the 48 cases with negative all five conventional *H. pylori* tests, 35 had no gastritis and 13 had gastritis. Among 13 *H. pylori*-negative gastritis (HpN) patients, two were *H. pylori*-positive by 16s rRNA gene sequencing and were excluded. Similarly, 11 samples from the control group had detectable levels of *H. pylori* by 16S rRNA sequencing and were excluded.

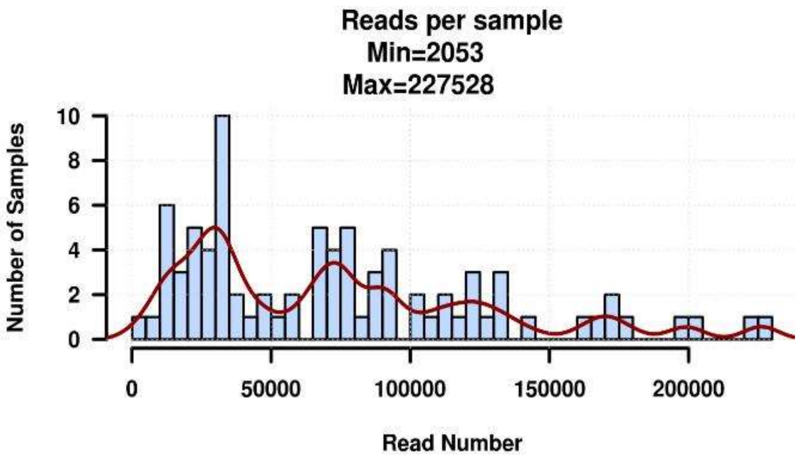

Figure S2. Overall sequence reads. Number of sequence reads per samples were shown.

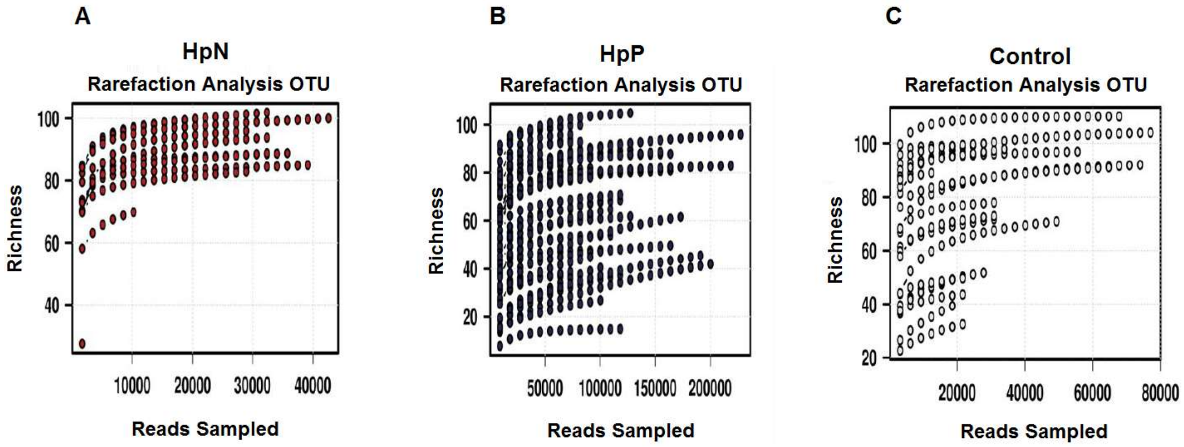

Figure S3. Rarefaction analysis. Rarefaction analysis is shown in comparison groups separately: HpN (**A**), HpP (**B**) and Control (**C**).

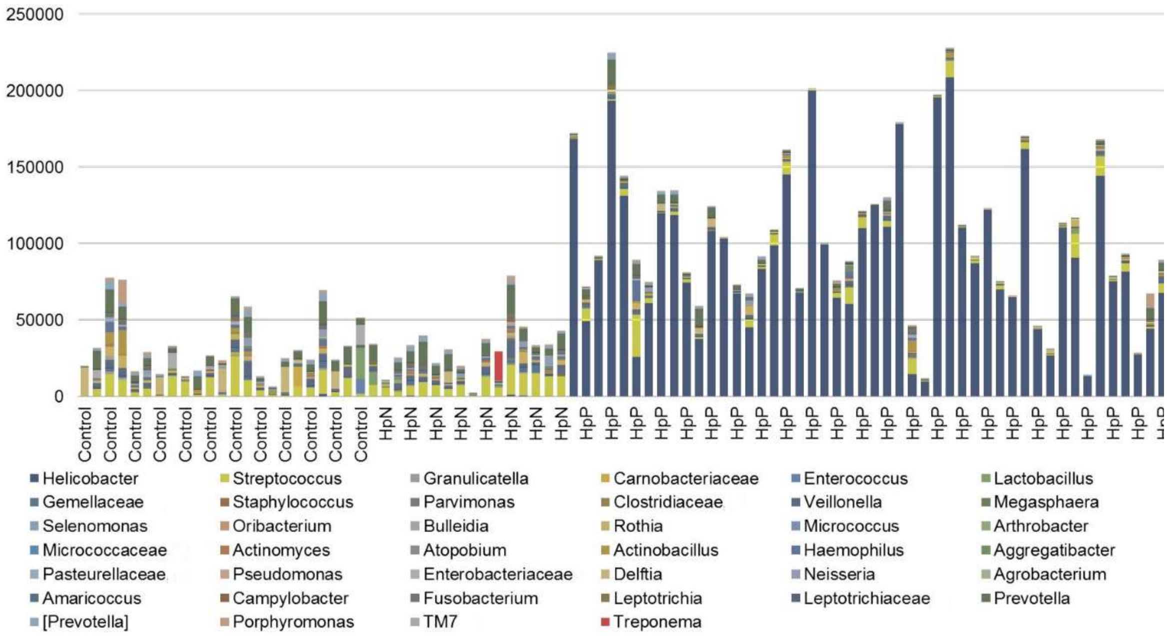

(**A**)

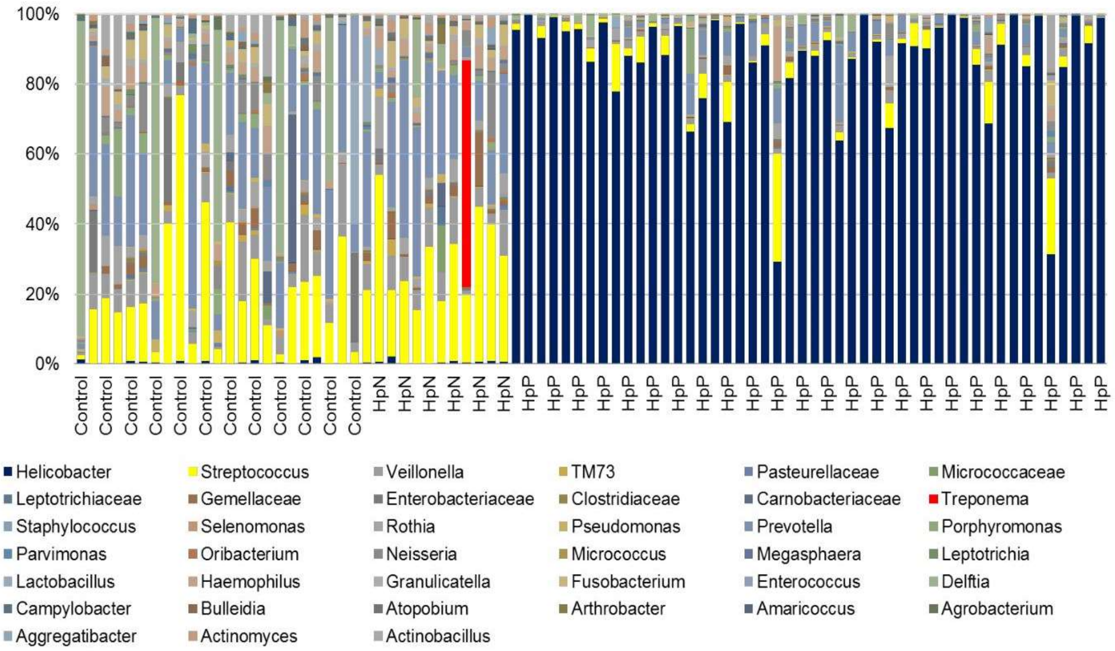

(**B**)

Figure S4. Bacterial relative abundance per sample. The overall bacterial reads based on raw read counts after filtering for low abundant OTUs (**A**) and their relative abundance (**B**) are shown in each comparison groups at genus level per sample.

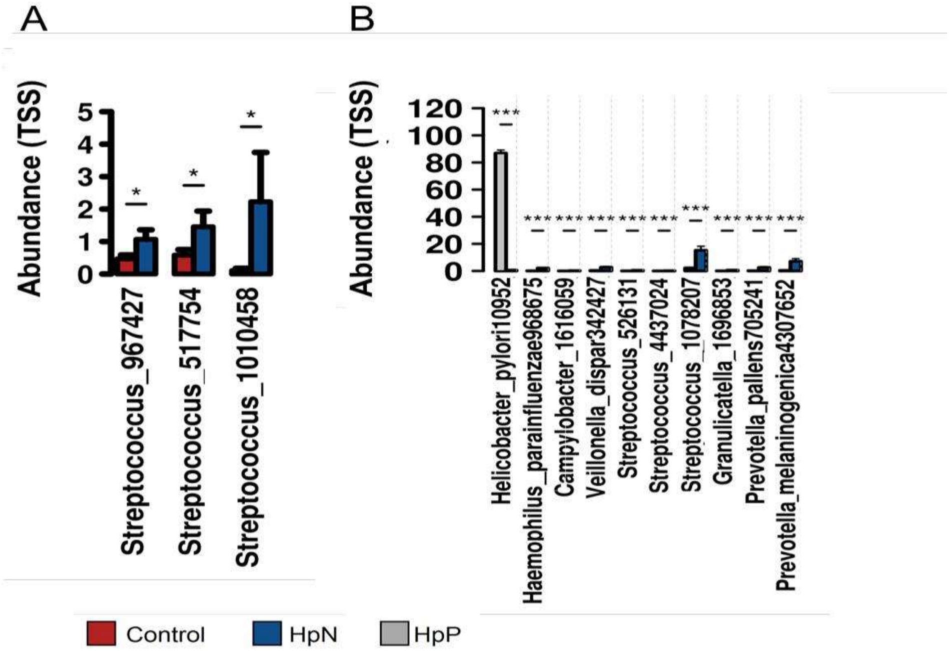

Figure S5. ANOVA test comparisons for bacterial abundance. Bacterial abundance differences were observed for 3 species in HpN vs control group (**A**), 48 species were significantly different between HpN and HpP group and the top 10 species are shown (**B**). TSS: Total -sum normalization. *: $p < 0.05$, ***: $p < 0.001$.

A

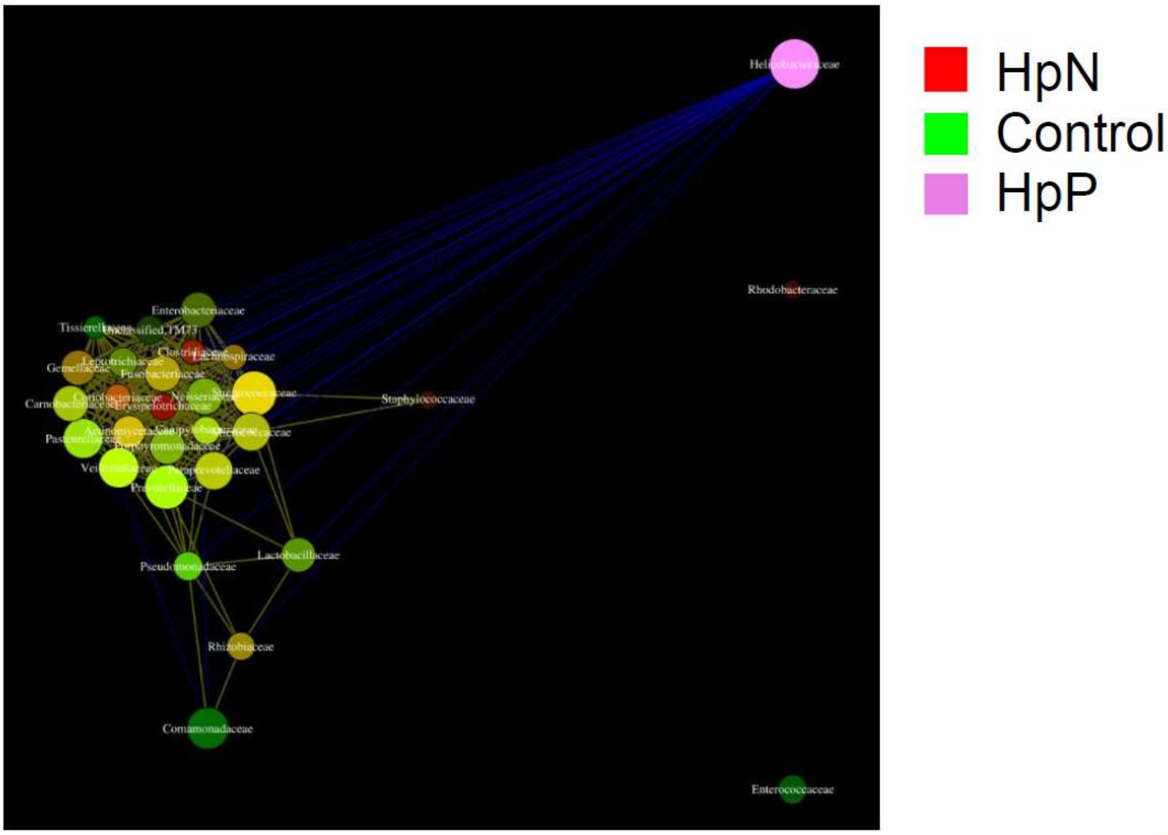
HpN
Control
HpP
Rhodobacteraceae
Enterobacteriaceae
Staphylococcaceae
Lactobacillaceae
Pseudomonadaceae
Rhizobiaceae
Comamonadaceae
Enterococcaceae

B

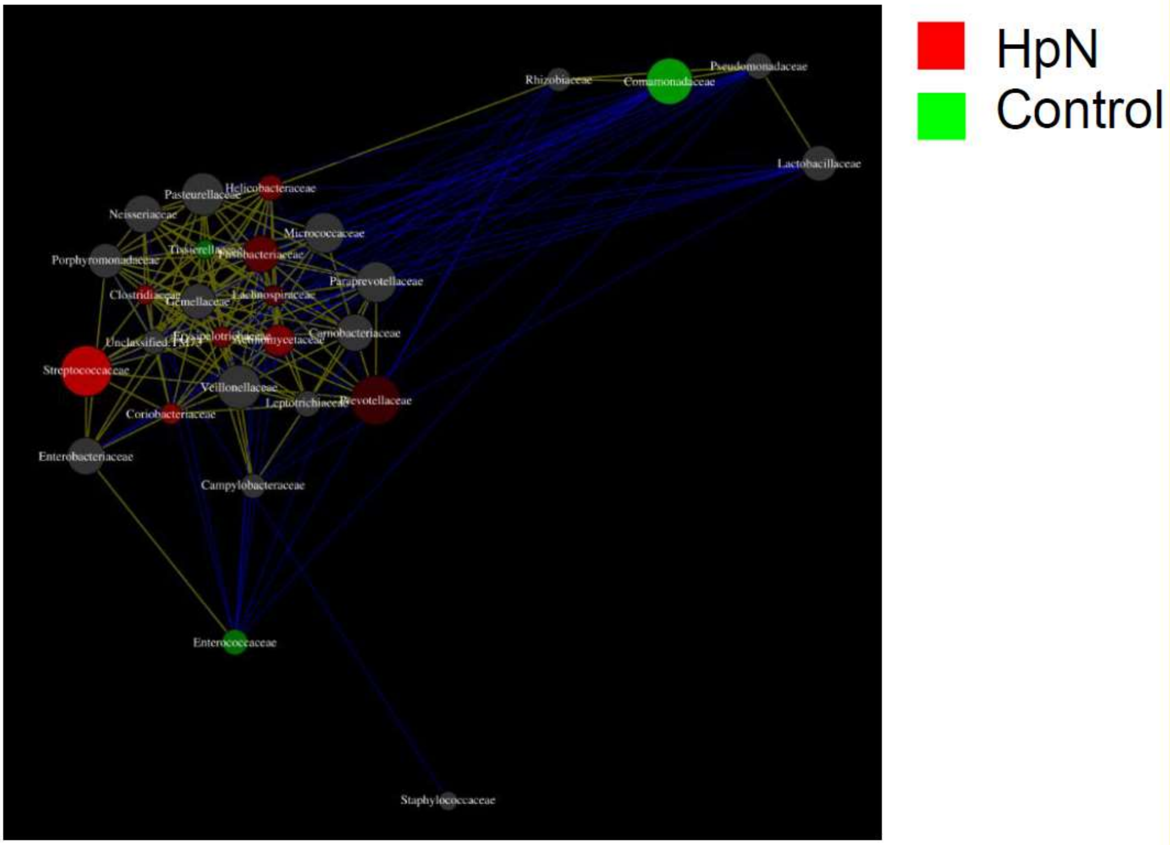
HpN
Control
Rhizobiaceae
Comamonadaceae
Pseudomonadaceae
Lactobacillaceae
Helicobacteraceae
Pasteurellaceae
Neisseriaceae
Micrococcaceae
Porphyromonadaceae
Paraprevotellaceae
Streptococcaceae
Veillonellaceae
Prevotellaceae
Coriobacteriaceae
Enterobacteriaceae
Campylobacteraceae
Enterococcaceae
Staphylococcaceae

C

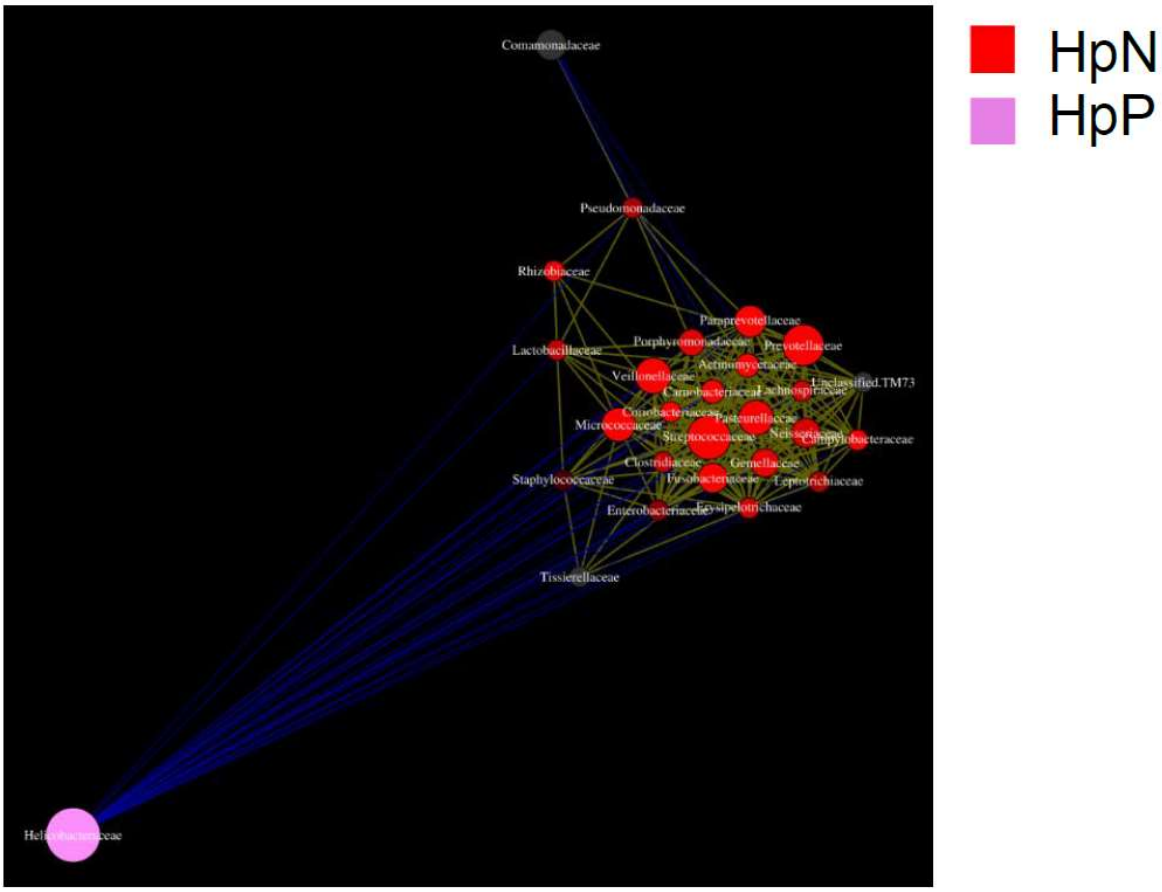

Figure S6. Network analysis for comparison groups A. The co-occurrence and disease specific bacterial interaction according to overall group (**A**), HpN vs. HpP group (**B**) and HpN vs. control group (**C**) were shown at family level. Taxa are represented as nodes, taxa abundance as node size, and edges represent positive associations (positive correlations between nodes are presented in yellow, negative correlations in blue). Nodes can be colored based on their association with selected environmental variables based on Pearson's correlation.

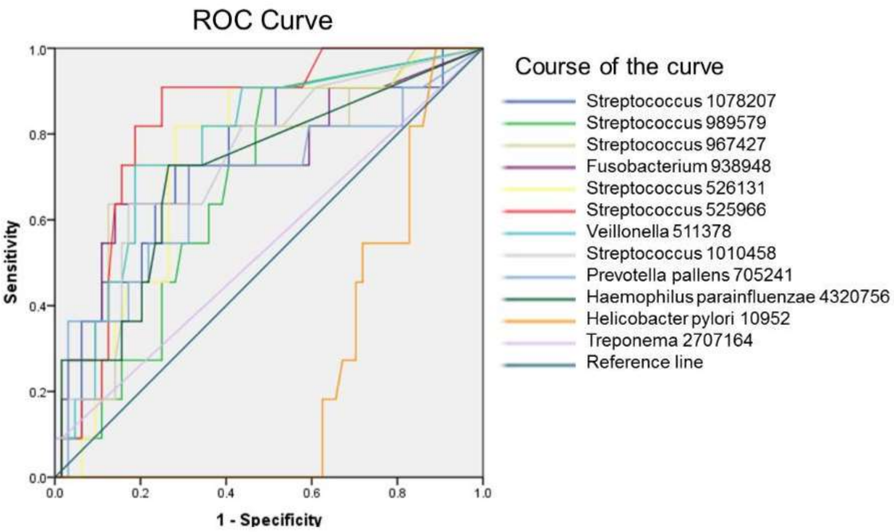

Figure S7. Receiver operating curve analysis for selected microbial biomarkers of *H. pylori* negative gastritis. 11 selected microbial biomarkers of *H. pylori* negative gastritis were tested by ROC analysis. In addition, *H. pylori* was included.

Table S1. Age and gender status according to the three study groups.

Disease	Mean Age ± SD	Male to Female Ratio
Control	48.5 ± 17.9	0.5
HpN	52 ± 17.1	0.6
HpP	44.4 ± 13.6	0.3
p value	0.3 †	0.5 ‡

† ANOVA, ‡ Chi square.

Table S2. Selected candidates for microbial biomarkers in *H. pylori* negative gastritis.

	Genus	Species	*p* Value	AUC	AUC Lower CI	AUC Upper Upper CI	OR	OR Lower CI	OR Upper CI
HpN vs. HpP and control group	Streptococcus	967427	0.00016	0.87	0.72	1	22.8	4.08	189.89
	Streptococcus	989579	0.00044	0.83	0.69	0.98	21.58	4.34	139.67
	Streptococcus	1010458	0.00023	0.84	0.71	0.98	8.67	0.75	199.09
	Streptococcus	1078207	0.00002	0.92	0.84	1	50.67	8.65	472.54
	Streptococcus	525966	0.00011	0.87	0.72	1	21.58	4.34	139.67
	Streptococcus	526131	0.00012	0.88	0.72	1	31.5	6.14	253.12
	Prevotella	pallens 705241	0.00021	0.87	0.7	1	68.25	9.19	1466.12
	Haemophilus	parainfluenzae 4320756	0.000079	0.84	0.69	0.99	22.29	2.81	472.84
	Fusobacterium	938948	0.0024	0.8	0.62	0.98	14.8	2.99	91.29
	Veillonella	511378	0.00063	0.83	0.65	1	68.25	9.19	1466.12
HpN vs. HpP group	Actinomyces	1089121	0.0042	0.78	0.57	1	46.8	6.35	988.54
	Actinomyces	12574	0.00077	0.83	0.68	0.98	4	0.82	19.3
	Actinomyces	875735	0.00017	0.87	0.72	1	46.8	6.35	988.54
	Atopobium	4451251	0.00011	0.87	0.72	1	104	13.46	2325.44
	Rothia	dentocariosa 4304654	0.0092	0.75	0.55	0.95	14.62	1.64	317.96
	Rothia	mucilaginosa 1017181	0.003	0.8	0.62	0.97	10.8	2.34	57.66
	Rothia	mucilaginosa 866280	0.0032	0.79	0.59	0.99	14.8	2.99	91.29
	Porphyromonas	970138	0.037	0.7	0.52	0.89	4.72	1.07	21.4
	Prevotella	4304901	0.004	0.76	0.6	0.93	10.86	1.78	90.35
	Prevotella	4321398	0.00021	0.84	0.68	1	46.8	6.35	988.54
	Prevotella	4324196	0.0035	0.79	0.61	0.97	15.83	2.77	130.53

	Prevotella	4426163	0.0064	0.75	0.57	0.92	10.28	2.02	62.37
	Prevotella	4458304	0.0015	0.81	0.65	0.97	15.83	2.77	130.53
	Prevotella	530206	0.016	0.74	0.52	0.95	14.8	2.99	91.29
	Prevotella	851822	0.003	0.79	0.59	0.99	46.8	6.35	988.54
	Prevotella	melaninogenica 4306852	0.0012	0.82	0.66	0.97	21.58	4.34	139.67
	Prevotella	melaninogenica 4307391	0.0038	0.78	0.6	0.97	10.86	1.78	90.35
	Prevotella	melaninogenica 4307652	0.000019	0.93	0.83	1	68.25	9.19	1466.12
	Prevotella	melaninogenica 525942	0.0094	0.75	0.56	0.94	8.4	1.91	41.25
	Prevotella	melaninogenica 535359	0.029	0.71	0.52	0.91	5.14	1.01	27.09
	Prevotella	nanceiensis 4310398	0.041	0.7	0.52	0.88	1.56	0.2	8.65
	Prevotella	tannerae 2714267	0.012	0.7	0.52	0.89	15.83	2.77	130.53
	Clostridiaceae	527630	0.0076	0.75	0.56	0.93	10.28	2.02	62.37
	Gemellaceae	1074210	0.0026	0.78	0.58	0.98	21.58	4.34	139.67
	Gemellaceae	535353	0.00077	0.82	0.64	1	32.5	4.32	683.99
	Gemellaceae	858896	0.014	0.74	0.54	0.94	7.05	1.3	43.14
	Bulleidia	moorei 851938	0.0032	0.79	0.63	0.95	14.8	2.99	91.29
	Granulicatella	1696853	0.00007	0.89	0.74	1	32.89	6.35	236.41
	Megasphaera	4296242	0.0035	0.78	0.6	0.97	10.28	2.02	62.37
	Oribacterium	749837	0.0038	0.77	0.57	0.97	46.8	6.35	988.54
	Selenomonas	4307790	0.04	0.7	0.5	0.89	2.69	0.58	11.79
	Streptococcus	1079866	0.0025	0.79	0.61	0.97	8.4	1.91	41.25
	Streptococcus	1082539	0.0066	0.76	0.56	0.95	15.83	2.77	130.53
	Streptococcus	2024840	0.00029	0.86	0.69	1	40.5	7.55	342.18
	Streptococcus	3449122	0.0025	0.78	0.59	0.96	10.86	1.78	90.35
	Streptococcus	4307484	0.00014	0.87	0.78	0.97	2.74	0.33	19.08
	Streptococcus	4437024	0.00013	0.87	0.73	1	33.25	5.92	286.68
	Streptococcus	509773	0.0014	0.81	0.66	0.97	12.25	2.77	64.1
	Streptococcus	516966	0.011	0.75	0.6	0.9	8.67	0.75	199.09
	Streptococcus	517754	0.0026	0.8	0.6	1	10.28	2.02	62.37
	Streptococcus	521996	0.00018	0.87	0.71	1	50.67	8.65	472.54
	Streptococcus	538185	0.00013	0.88	0.72	1	21.58	4.34	139.67
	Veillonella	1023075	0.0065	0.75	0.59	0.92	4.62	0.74	29.41
	Veillonella	dispar 342427	0.00022	0.87	0.73	1	68.25	9.19	1466.12
	Veillonella	dispar 4310208	0.017	0.74	0.54	0.93	10.8	2.34	57.66
	Veillonella	dispar 4401373	0.00028	0.86	0.7	1	22.8	4.08	189.89
	Veillonella	dispar 4408758	0.00024	0.86	0.74	0.99	7.13	1.03	61.36

	Veillonella	dispar 962249	0.0017	0.81	0.65	0.96	5.83	1.29	27.96
	Fusobacterium	4313722	0.0017	0.8	0.68	0.93	4.63	0.74	29.41
	Pasteurellaceae	3825935	0.00022	0.85	0.71	0.99	10.86	1.78	90.35
	Actinobacillus	parahaemolyticus 971906	0.0049	0.76	0.57	0.94	7.05	1.3	43.14
	Aggregatibacter	4335776	0.016	0.72	0.52	0.92	22.29	2.81	472.84
	Aggregatibacter	segnis 92230	0.0072	0.74	0.56	0.93	15.83	2.77	130.53
	Agrobacterium	521073	0.00055	0.84	0.7	0.98	32.5	4.32	683.99
	Campylobacter	1616059	0.000014	0.92	0.78	1	104	13.46	2325.44
	Haemophilus	517548	0.00022	0.86	0.71	1	4.62	0.74	29.41
	Haemophilus	995893	0.0045	0.75	0.55	0.94	14.62	1.64	317.96
	Haemophilus	influenzae 240754	0.0000071	0.82	0.68	0.97	22.8	4.08	189.89
	Haemophilus	parainfluenzae 1671681	0.011	0.73	0.53	0.94	68.25	9.19	1466.12
	Haemophilus	Parainfluenzae 968675	0.000028	0.92	0.75	1	104	13.46	2325.44
	Helicobacter	pylori10951	0.0000005	1	1	1	0.01	0	0.04
	Neisseria	355750	0.017	0.72	0.54	0.9	7.05	1.3	43.14
HpN vs. control	Streptococcus	1010458	0.012	0.76	0.59	0.94	5.11	0.44	118.36
	Streptococcus	1078207	0.045	0.72	0.52	0.91	4.56	1	22.97
	Streptococcus	526131	0.048	0.71	0.52	0.9	4.56	1	22.97
	Streptococcus	967427	0.05	0.71	0.51	0.91	3.6	0.81	17.24
	Streptococcus	989579	0.057	0.7	0.52	0.88	3.6	0.81	17.24
	Streptococcus	525966	0.078	0.69	0.49	0.88	2.02	0.45	9.12
	Haemophilus	Parainfluenzae 4320756	0.0093	0.76	0.59	0.93	4	0.72	24.96
	Fusobacterium	938948	0.082	0.7	0.49	0.88	3.6	0.81	17.24
	Veillonella	511378	0.075	0.7	0.5	0.88	2.91	0.67	13.49
	Prevotella	Pallens 705241	0.084	0.7	0.48	0.89	3.6	0.81	17.24

© 2019 by the authors. Licensee MDPI, Basel, Switzerland. This article is an open access article distributed under the terms and conditions of the Creative Commons Attribution (CC BY) license (http://creativecommons.org/licenses/by/4.0/).
